# Coral Skeletons Defend against Ultraviolet Radiation

**DOI:** 10.1371/journal.pone.0007995

**Published:** 2009-11-25

**Authors:** Ruth Reef, Paulina Kaniewska, Ove Hoegh-Guldberg

**Affiliations:** Centre for Marine Studies and the Austrailan Research Council (ARC) Centre of Excellence for Coral Reef Studies, The University of Queensland, St Lucia, Queensland, Australia; University of Queensland, Australia

## Abstract

**Background:**

Many coral reef organisms are photosynthetic or have evolved in tight symbiosis with photosynthetic symbionts. As such, the tissues of reef organisms are often exposed to intense solar radiation in clear tropical waters and have adapted to trap and harness photosynthetically active radiation (PAR). High levels of ultraviolet radiation (UVR) associated with sunlight, however, represent a potential problem in terms of tissue damage.

**Methodology/Principal Findings:**

By measuring UVR and PAR reflectance from intact and ground bare coral skeletons we show that the property of calcium carbonate skeletons to absorb downwelling UVR to a significant extent, while reflecting PAR back to the overlying tissue, has biological advantages. We placed cnidarians on top of bare skeletons and a UVR reflective substrate and showed that under ambient UVR levels, UVR transmitted through the tissues of cnidarians placed on top of bare skeletons were four times lower compared to their counterparts placed on a UVR reflective white substrate. In accordance with the lower levels of UVR measured in cnidarians on top of coral skeletons, a similar drop in UVR damage to their DNA was detected. The skeletons emitted absorbed UVR as yellow fluorescence, which allows for safe dissipation of the otherwise harmful radiation.

**Conclusions/Significance:**

Our study presents a novel defensive role for coral skeletons and reveals that the strong UVR absorbance by the skeleton can contribute to the ability of corals, and potentially other calcifiers, to thrive under UVR levels that are detrimental to most marine life.

## Introduction

Photosynthesis is a common pervasive characteristic of shallow tropical marine habitats with organisms being photosynthetic or involved in a tight symbiosis with photosynthetic symbionts. In the latter case, the intimate association of animals such as corals and these primary producers plus the efficient recycling of nutrients underpins their success in the generally nutrient poor waters of the tropics. In this respect, reef-building corals rely greatly on photosynthates produced by their symbiotic photosynthetic dinoflagellate, *Symbiodinium* [1], which can harnesses the abundant solar energy in the tropics to fix carbon and translocate organic carbon for coral respiration [2]. In return, *Symbiodinium* gains access to the inorganic nutrients flowing from the catabolic processes of the coral host. The autotrophic energy provided by *Symbiodinium* to the coral host results in carbon fixation by coral reefs that is six times higher than that in neighbouring oligotrophic waters [3], [4], allowing for the formation of complex reef structures which provide niches for a diverse range of organisms.

The symbiosis between scleractinian corals and *Symbiodinium* probably arose in the late Triassic [5]. Corals have evolved to optimise the photosynthetic activities of the resident *Symbiodinium* through changes to their morphologies [6], [7], [8] or through changes in tissue composition [9] or population density of *Symbiodinium* [10], [11]. As a result of these evolutionary pressures, corals have evolved into highly efficient light-harvesting organisms [12]. They can utilise light six times more efficiently than plants [10] due to multiple scattering of photons within the skeleton and the tissue-water interface [13], thereby increasing photonic path lengths and subsequently the chance of interception by a photosystem [13]. This enhancement of Photosynthetically Active Radiation (PAR) allows the coral to increase its photosynthetic yields. However, as solar radiation also contains Ultraviolet Radiation (UVR), an increase in PAR could be accompanied with side effects of a considerable increase in harmful UVR.

UVR photons contain enough energy that upon absorption they break chemical bonds. The most sensitive of the organic molecules are aromatic compounds [14] such as DNA, proteins and membranes. Direct damage caused by the absorption of a UV photon by DNA can manifest in the formation of cyclobutane pyrimidine dimers (CPDs), which can make up 75% of UV-induced DNA lesions [15], 6–4 photoproducts (6–4PPs) or the Dewar valence isomer of the 6-4(PP). UV can also act indirectly and create lesions such as oxidised or hydrated bases, single-strand breaks and more [16]. CPDs, the greater part of the DNA damage observed and the focus of our study, are formed between two adjacent pyrimidine bases in DNA exposed to UVR and are known to induce cell death [17], [18]. Thus, while exposure to solar radiation is fundamental for coral growth, avoiding UVR damage is just as vital.

Around 15% of net reef productivity is used to generate the carbonate skeletons of corals, which ultimately results in the reef framework [3]. Calcium carbonate skeletons serve multiple roles such as protection and structural strength, and the highly reflective white skeleton can scatter light back into the overlying tissue, increasing the chance of photons interacting with the photosynthetic *Symbiodinium* [10], [13]. The coral skeleton is extracellular and located at the base of coral tissue. The skeleton is made out of calcium carbonate (CaCO_3_) crystallised in aragonite (orthorhombic system) along with minute amounts of organic matter (<0.1% of total weight) [19] and trace metals [20], [21].

The discovery of fluorescent banding in coral skeletons irradiated by UVR [22], has led to numerous studies utilizing this phenomenon in the study of sclerochronology [23]. But despite its frequent use as a record of past processes and events, why skeletal fluorescence occurs is not well understood. Emissions from coral skeletons irradiated by UVR involve both fluorescence and phosphorescence [24]; and will be referred to hereafter as luminescence. While generally believed to be related to environmental conditions, higher luminescence does not necessarily correlate with skeletal density [25], and there is considerable debate over whether it correlates with weather patterns [26], [27].

Luminescent bands are prevalent in many coral species, through the geological records, at inshore and offshore reefs and at different locations worldwide. Luminescence in corals can result from both organic and inorganic sources. Organic luminescence stems primarily from the incorporation of naturally luminescent humic acids, the major constituent of dissolved organic matter (DOM) in the sea [28]. Inorganic luminescence comes from the chemical properties of the carbonate skeleton. An intrinsic blue luminescence has been observed in carbonates, including aragonite, but it is weak and overshadowed by extrinsic luminescence when activator elements are incorporated into the crystal [29]. Transition metal ions occupy the Ca^2+^ site of the carbonate and this results in a strong yellow luminescence [30]. Such inorganic luminescence occurs in corals when trace elements are incorporated in the coral skeleton (such as Mg, Mn, Zn, Sr and Cu). Though not luminescent, ferric iron absorbs strongly in the UV and can also be a common trace element in coral skeletons [31].

We have been examining the luminescent properties of coral skeletons and hypothesise that these properties of coral skeletons can serve as a UV defence protecting the overlying coral tissue by reducing UVR levels that might otherwise have been amplified by the highly reflective coral skeleton. We suggest that the absorption of the UVR photon by the skeleton and its remission in a “safe” longer wavelength can result in the reduction of the amount of UVR that the coral tissue is exposed to. We tested this hypothesis by placing symbiotic anemones (which have similar tissue properties to those of symbiotic corals) on top of coral skeletons and measured the incidence of UV inflicted DNA damage they accumulated in comparison with anemones grown on a substrate that reflected UVR as well as PAR.

## Results

**Figure 1:**
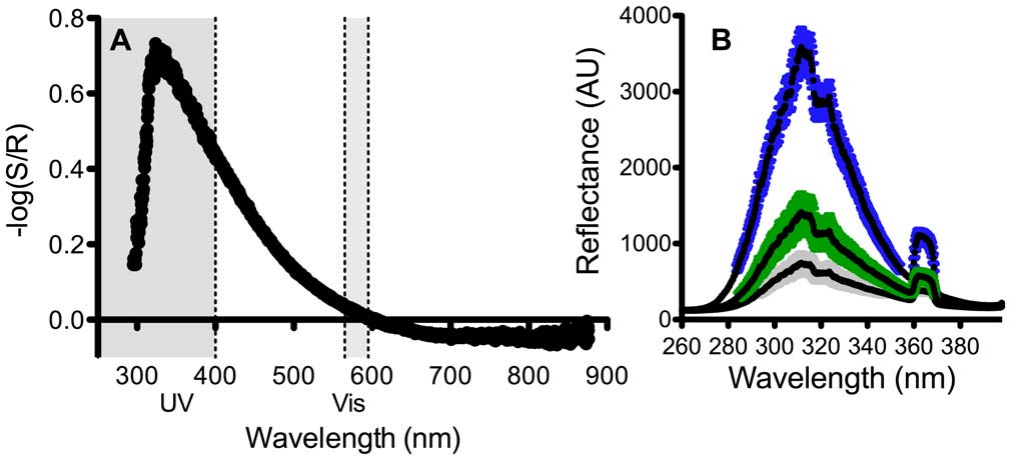
Derived absorbance of the coral skeleton. A) Derived absorbance spectrum of the white *Echinopora sp.* skeleton under full sunlight. Shaded areas correspond to the UVR (250–400 nm) and visible (558–595 nm) peaks used in this study. Maximal absorption occurred at 330 nm. R is reflectance measured from the PTFE reflector and S is reflectance from the skeleton. Slightly negative absorbance values >600 nm are due to a lower reflectivity of PTFE in that range (Fig. 5). B) Average (*n* = 10) reflectance of downwelling UVR (AU, arbitrary units) measured from PTFE (blue), *Stylophora pistillata* skeletons (green) and *S. pistillata* skeletons crushed to a fine powder (grey). Error bars are standard deviations from the mean.

**Figure 2:**
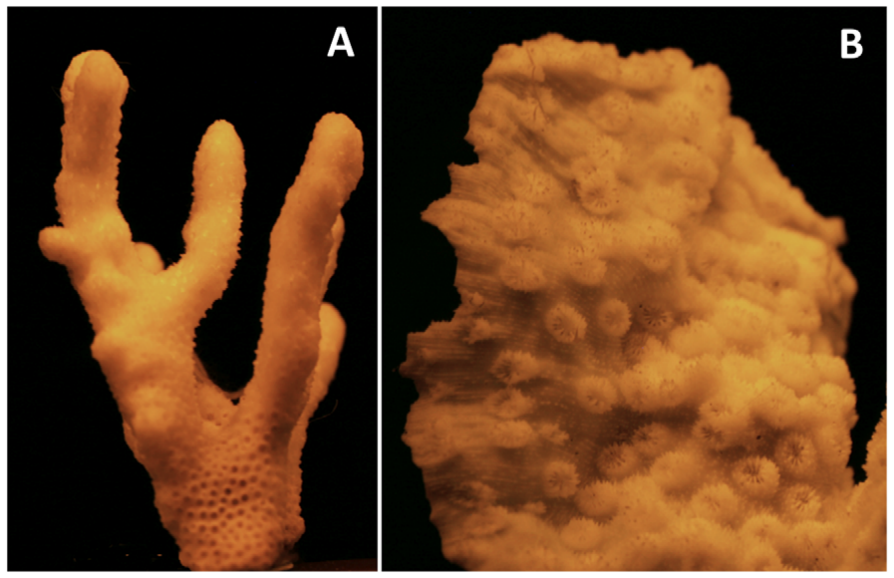
Luminescence of the coral skeleton. A) A long exposure photograph of a *Stylophora pistillata* skeleton irradiated with mid range UVR, photographed through a barrier UV filter, showing characteristic yellow fluorescence A photograph of an (B ..skeleton taken in the same manner .sp Echinopora.

**Figure 3:**
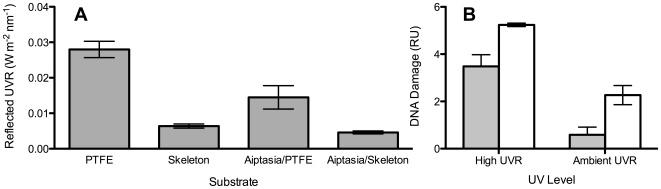
Defence from UVR in overlying tissue. A) Mean (*n* = 3) and SD of UVR reflected from PTFE, Skeleton, anemones on top of PTFE and anemones on top of a skeleton (see Fig. 6 for schematic) in W m^−2^ nm^−1^, PTFE reflected 4 times more UVR than the skeleton, resulting in a significantly lower transmittance of UVR through the anemone tissues on top of the skeleton compared with those on top of the PTFE. B) DNA damage in the form of CPDs (mean±SEM, *n* = 6) in relative units (RU) for *Aiptasia pulchella* on PTFE (white bars) and coral skeleton (grey) as a substrate under ambient and high UVR levels. *A. pulchella* placed on top of a skeleton had significantly fewer CPDs than those placed on top of PTFE, under both ambient and high UVR levels.

While being highly reflective for photosynthetically active radiation, coral skeletons do not reflect UVR to any real extent (Figs. 1A, 1B). Grinding the skeletons to a fine powder did not reduce the average UVR reflectance of skeletal material (paired t-test, p>0.8, n = 10; Fig. 1B), indicating that the UV luminescence is a fundamental property of coral aragonite and not due to structure larger than 100 µm. Skeletons illuminated with UVR (280–360 nm) showed a weak yellow fluorescence (Figs. 2A, 2B). The substrate material (skeleton or polytetrafluoroethylene, PTFE) had a highly significant affect on the UV reflectance (One-Way ANOVA, F(3,8) = 82, p<0.001, Fig. 3A). The level of UVR reflected from PTFE was 4 times higher than that reflected from the *Echinopora* sp. skeleton (Tukey HSD, p<0.001). The UV reflected from anemones placed on PTFE was significantly higher than that from anemones placed on top of skeletons (Tukey HSD, p = 0.001), indicating that less UVR passed through the tissue of anemones placed on top of the skeleton compared to their counterparts placed on top of the UV reflective PTFE.

There were significantly fewer CPDs in the anemones placed over skeletal material under both ambient and high UVR compared to the damage levels in anemones placed on top of PTFE tape (Fig. 3B). UV-inflicted DNA damage was seven times higher in anemones on top of the PTFE reflector than in those placed on top of the coral skeleton when exposed to UVR levels of shallow coral reef environments (Factorial ANOVA F(1,16) = 49.4, p<0.001). Under higher doses of UVR, UV-inflicted DNA damage was overall higher (F(1,16) = 445.5, p<0.001), but remained lower in anemones placed on top of skeletons compared to those on top of PTFE. We did not detect a significant interaction between substrate type and UV level on resulting DNA damage indicating that damage was reduced under both ambient and high UVR levels over the skeleton compared to the reflector.

The substrate material had no significant affect on visible light reflectance (One-Way ANOVA, F(3,8) = 3.88, p>0.05, Fig. 1A). The reflected visible light from the skeleton was on average 20% less compared to the PTFE reflector, but the difference was not found to be statistically significant. The reflected visible light through the anemones was 60% that reflected from the adjacent substrate (skeleton or PTFE), indicating significant absorbance of visible light by the anemone tissue.

## Discussion

Reef-building corals face a dilemma akin to that of a “Catch 22” [sensu 32]. While being exposed to full solar radiation is advantageous in terms of their energy budget, it has very negative consequences in terms of increased exposure to harmful UVR. We have shown that coral skeletons have high absorbance in UVR, emitting a weak yellow fluorescence as a result. This is consistent with observations of previous studies [e.g. 22], [33]. By emitting potentially harmful UVR as safe yellow light, coral skeletons dampen the amplification of the UVR while amplifying and increasing harvesting efficiency of the PAR that is necessary for growth and survival [10], [12]. In this respect, coral skeletons reflected PAR at a level that was similar to reflective white PTFE tape.

Cnidarian tissue were exposed to significantly lower levels of UVR when placed on top of a bare coral skeleton as opposed to a white reflector. Had the coral skeleton reflected UVR as well as it does visible light, the overlying tissues would be exposed to significantly higher levels of lethal UVR. The extent to which the skeleton reduces UVR in overlying tissues of live corals is hard to predict as the attachment of coral tissue to the skeleton is complex and placing *Aiptasia* on top of coral skeletons did not simulate this attachment fully. Nonetheless, it is clear from our results that skeletons reduce UVR in overlying tissues to a significant extent. The ability to uncouple PAR and UVR appears to be crucial to the success of corals in the highly sunlit waters of the tropics and explains how corals have evolved to trap light so efficiently.

Corals are just one group of marine calcifiers. Calcification spans kingdoms and carbonate skeletons are deposited by stromatoporids, echinoderms, most molluscs, algae, foraminifera, cocolithophores and many others. There is a strong link between photosynthesis and calcification. The majority of the organisms that deposit aragonite or calcite skeletons photosynthesise [34]. These organisms would favour high PAR environments and would have to remain exposed to PAR, and consequently to harmful UVR in order to survive. For this reason, it is possible that possessing calcium carbonate skeletons might impart advantages in terms of uncoupling of the exposure of tissues to PAR and UVR. While speculative at this point, further exploration of this potential evolutionary driver of calcium carbonate skeletons is warranted in our opinion.

In most organisms studied previously, the potential functions of UV luminescence in animals are not well understood, and are usually attributed to social and behavioural cues [35]. Similarly to coral skeletons, scorpion cuticles fluoresce at the visible spectrum (400–700 nm) if irradiated by UV light [36], [37] as do wasps [38], spiders [39] and mantis shrimp [35]. Frost et al. [40] have suggested that in scorpions, luminescence might have provided survival advantage, serving as a sun block, a relict feature from when scorpion habits were more diurnal. Fisher [41] proposed that biomineralisation served to defend pre-Cambrian cyanobacterial mats from UVR. Here we provide the first evidence supporting these hypotheses.

The skeleton greatly reduced the amount of UVR in the tissue above it but it did not eliminate UVR altogether. Furthermore, the skeleton is deposited below the tissue, so UVR photons must pass through the tissue at least once, ensuing other methods must be employed by the coral to protect against UVR damage. Corals deal with UVR in many ways. One mechanism to defend their tissues from UVR is to use sunscreen molecules, (mycosporine like amino-acids, MAAs [42], [43]). This pathway, however, requires a large energy input to produce and maintain the pigment molecules involved [44]. Corals also have an efficient DNA repair mechanism that rapidly targets UV-inflicted DNA damage when it occurs [18]. In combination, these pathways provide a high level of protection against UVR and contribute to the ability of corals to thrive under ultraviolet levels that can be lethal to other coral reef epifauna [45].

We found that grinding the skeleton to a fine powder did not significantly change its absorbance properties, leading us to believe that the absorbance is mostly a property of the skeletal material and not dependant on structure greater than 100 µm. Organic luminescence such as from humic acids can be incorporated into the skeleton during periods of terrestrial runoff [28], [46]. Strongly UV absorbent, iron-rich clay is common in the ocean, even in clear tropical waters and minute quantities of clay can be incorporated into the coral's aragonite skeleton [47], this too can be dependent on sediment input. Olson and Pierson [48] have demonstrated that inorganic iron, when present in the soil even in minute quantities, can provide UV protection to the mat forming phototrophic bacteria *Chloroflexus aurantiacus*. Inorganic luminescence can be dependant on skeletal density [33] and on changes to the skeletal chemistry. Incorporation of trace elements into coral skeleton affects its luminescent properties and is dependant on both ambient water conditions (such as temperature and pH) [49], [50] and physiological processes of the coral [51], [52]. The newly discovered function of the skeleton as a UV defence for the overlying tissue denotes that any changes to skeletal luminescence, due to changes in skeletal density, chemical composition or incorporation of organic substances can potentially change coral sensitivity to UVR.

## Materials and Methods

### Reflectance Measurements

**Figure 4:**
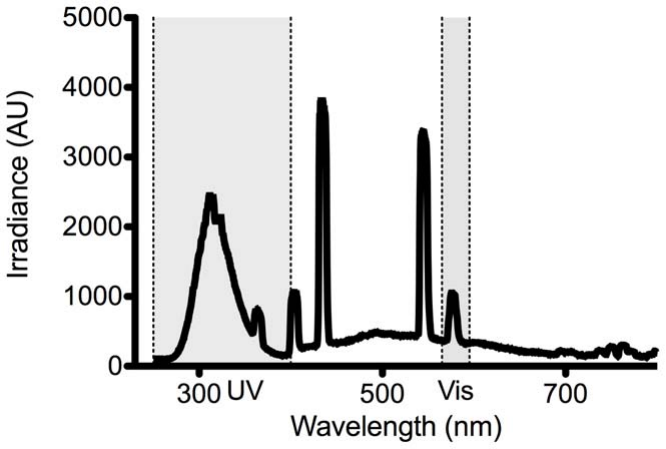
UV lamp spectrum. Downwelling irradiance (in arbitrary units) measured at the skeleton surface from the 4 W UV-B lamps used in this study. Shaded areas correspond to the UV peak (250–400 nm) and the visible peak (558–595 nm) used in this study.

**Figure 5:**
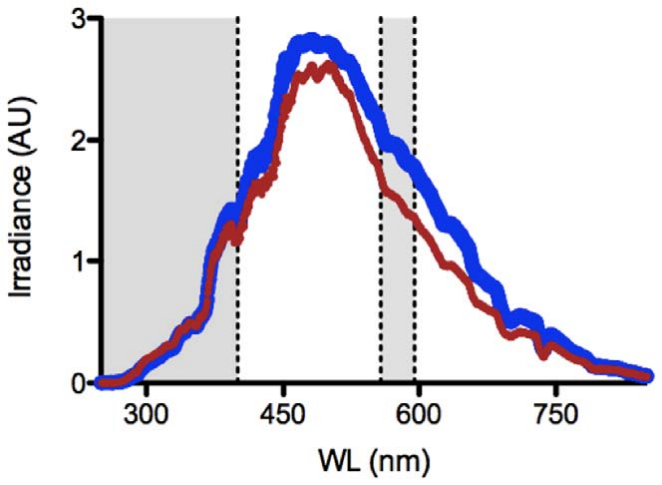
PTFE reflectance. Natural solar irradiance in arbitrary units reflected off PTFE (red) compared with downwelling irradiance (blue) measured between 200–850 nm at 0.3 nm intervals using a USB2000 spectrometer (Oceanoptics, USA). In the UV region a one to one correlation was found and over the visible wavelengths, reflectance was >95%. Shaded areas correspond to the UV peak (250–400 nm) and the visible peak (558–595 nm) used in this study.

UV radiation was emitted from a 4W Sankyo G4T5E UV-B lamp (Sankyo Denki Co. Ltd. Kanagawa, Japan), which emits UVB as well as some visible light (Fig. 4). Reflectance was measured using a USB2000 spectrometer (Ocean Optics, Dunedin, FL, USA) with a bandwidth of 200–850 nm and an attached optic fibre with a core diameter of 1000 µm. The sensing end of the fibre-optic was held at a constant distance of 2 cm above the surface, positioned so as not to cause self-shading. Reflected light spectra from the coral skeleton were compared to those from a reflector at the same distance and angle. We used white polytetrafluoroethylene tape as a reflector (PTFE, or plumbing tape). The reflectance of this material was >99% between 250–800 nm, but slightly lower in the visible range (only ∼95%, Fig. 5). Reflectance measurements were made on intact and finely ground (<100 µm) *Stylophora pistillata* skeletons. In addition, reflectance measurements of natural sunlight as well as of a UV lamp were made on an *Echinopora* sp. skeleton as described below.

### Determining the Influence of Coral Skeletal Structure on UV Absorbance

We measured UV reflectance from 10 intact *Stylophora pistillata* skeletons and repeated the measurement after crushing the skeletons to a fine powder (<100 µm) using a mortar and pestle. Reflectance was measured as described above. UV absorption was measured as the negative logarithm of reflectance from the skeleton divided by reflectance from the reflector. The data was tested using a two-tailed paired t-test.

### Testing the Effect of UV Fluorescence on the Overlaying Tissue

*Aiptasia pulchella*, a cryptic symbiotic tropical anemone were placed on top of a plating coral skeleton (*Echinopora* sp.). *Aiptasia* have been used as a model organism for cnidarian biology for over 30 years [53] and are very similar biologically to corals with the exception of the carbonate skeleton, as anemones lack a skeleton. Half of the coral skeleton was covered in three layers of white PTFE tape and half was left uncovered. Anemones were distributed evenly between the two halves and were anesthetised using 0.18 M MgCl_2_ in seawater to keep tentacles open and immobilise the anemones throughout the experiment. The PTFE tape simulated the high reflectance of the coral skeleton but over the entire spectrum (including UV). The anemones were placed under two different levels of UV-B light (ambient, 2.25 W m^−2^ and high, 4 W m^−2^) for 25 minutes, then snap frozen in liquid N_2_ and processed for DNA damage. Ambient UVR levels were comparable to those expected at the Great Barrier Reef from the libRadtran model [54] and were achieved by using one G4T5E UV-B lamp (Sankyo Denki Co. Ltd. Kanagawa, Japan), while high UVR was achieved by using two such lamps. In each experiment 3 anemones were placed on a skeleton and 3 were placed on a reflector, this was repeated twice (n = 6 for each treatment). Data was evaluated for normality and homogeneity of variance and a factorial ANOVA was performed to test for differences in DNA damage using R version 2.8.1 [55].

**Figure 6:**
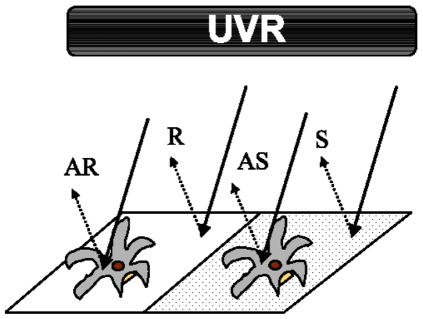
Schematic of the reflectance light measured from the different substrates. Reflectance collected and measured off a coral skeleton (S), a reflector (R), and the anemones on top of the skeleton (AS) and the reflector (AR). Measurements were made in the UV range (250–400 nm) as well as the visible (558–595 nm). The irradiance source was one or two 4 W UV-B lamps (Fig. 4).

An absorbance spectrum under full sunlight was calculated for the *Echinopora* sp. skeleton in order to determine its absorbance properties. Reflectance was measured from the skeleton (S) and the reflector (R) as above, both blanked against a black standard (which showed 0 reflectance throughout the spectrum) and the derived absorbance was calculated as −log(S/R) [56]. To understand why damage might be reduced over skeletal material, the reflective properties of the coral skeleton (S), the reflector (R), and the anemones on top of the skeleton (AS) and the reflector (AR; Fig. 6) under the UV lamp were measured for 3 anemones in each treatment with methods described above. UV reflectance was then calculated as the sum of reflected irradiance between 250–400 nm and was calculated by performing a cubic spline interpolation on the spectral irradiance data (*splinefun*, between 250–400 nm) followed by integration to get the area under the curve (AUC) [55]. For the visible light peak, the AUC was calculated between 558 and 595 nm (Fig. 4), in this range light absorption by the skeleton was not detected (Fig. 1A).

### Quantification of DNA Damage

DNA was isolated and damage quantified following the method described in [18]. Briefly, anemones were homogenised in extraction buffer (100mM EDTA, 10mM Tris, 1% SDS, pH 7.5) and incubated at 65°C for an hour. Proteinase K was added to a final concentration of 500 µg/ml and incubated at 37°C overnight. Samples were extracted once with phenol chloroform isoamyl alcohol (1∶24∶1), once with chloroform, then ethanol precipitated twice and redissolved in TE (0.01 M Tris-HCl, pH 7.5, 0.001 M EDTA). The different samples were all brought to a concentration of 0.02 ng µl^−1^ DNA in 1× PBS (Phosphate Buffer Saline). The DNA was then bound to standard ELISA plates and probed with the primary antibody TDM-2, which binds to the UV-induced CPDs present in the DNA [57]. Absorbance at 492 nm for each sample was read using a plate reader, with increased absorbance corresponding to increased amounts of DNA damage.
